# On the Treatment of Varicose Veins

**Published:** 1829-01-01

**Authors:** 


### LVII.

### Treatment of Varicose Veins.

A varicose affection of the veins, especially of those of the lower extremities, is so common, that its treatment is a matter of no small importance to the medical man, as well as to his patient. We do not intend to mention all the modes which have, at various times, been tried: suffice it to say, that a safe and a certain cure is still a very great desideratum. Blistering over the vein is an old, and, on the whole, a good-for-nothing plan; but, pushing the principle somewhat farther, Mr. Mayo recommends the application of caustic in the same situation.

"The method which I employ consists in applying potassa fusa, made into a paste with soft soap, to the integument covering the vein. I cut a hole, one-third of an inch in depth, and of the requisite length, (from an inch to two inches) in a piece of leather upon which adhesive plaister has been spread: the plaister is then applied to the skin, so that the length of the aperture is transverse to the vein or veins I would obliterate. The hole in the plaister is then filled with the caustic paste; and a piece of adhesive plaister, and a roller applied over *that*, prevent its shifting. In seven hours the roller, plaister, and paste, are moved, the part washed with warm water, and a linseed poultice applied. In about ten days the slough produced by the action of the caustic separates; in a week to ten days more the sore is cicatrized, and the cavity of the vein is found to have become obliterated.

"For the first two days after the application of the caustic paste the adjoining part of the vein is hard and sore upon pressure: to relieve this, nothing has been necessary besides desiring the patient to remain at rest, with the leg on a sofa, to take opening medicine, and to live upon broth and tea, and to apply to the part the liquor plumbi dilutus, as a lotion. The flow of the blood through the vein has commonly ceased about the fifth or sixth day: sometimes I have found, on tapping with my hand the swollen vein below the caustic, that by the second day the fluctuation has ceased to be communicated to the blood in the part above. In a few instances, when the slough of the integument has separated *the vein has been seen as a second slough, traversing the bottom of the ulcer*. The vein always appears to be obliterated for some little distance above and below the part exposed to the action of the caustic.

"I have applied caustic thus upon the great saphena vein above the knee, but more commonly to the same vein below the knee; to a part evenly dilated, and across a knotted part; to the saphena minor, immediately below the knee, and to the saphena major, in two places at once, near the knee and near the ankle; it has never failed to obliterate the vein in any case which I have witnessed; no hæmorrhage has ever taken place; no local inflammation more than I have described; no symptomatic fever."* 813.

It would be useful to know in how

---

* Med. Gazette.

many cases this method has been tried; for we cannot but suspect it would prove very dangerous, if generallv used. Division of the vein with a valvular wound of the integument is surely no violent injury to the vessel, and yet it is occasionally followed by fatal inflammation; the ligature is notoriously so dangerous, as now to be almost universally abandoned; and excision is almost as much to be dreaded as the ligature. Looking at these facts, we dislike Mr. Mayo's proposal; because the inflammation excited by the caustic may, indeed positively *must*, be more severe than that which results from either division, excision, or the ligature. In many of the cases, we are told that the vessel is seen to *slough*—in all, it must inflame, and that pretty sharply. Perhaps it may be said, the plan has been tried with success in a certain number of cases: but the argument is good for nothing; for so was that worst of the other operations, the ligature. Sir Astley Cooper had run through a considerable part of his professional career before he met with one fatal case; but then they came crowding upon him, and induced him to declare, that he himself would rather have a ligature placed on his femoral artery, than upon his saphena vein. Sir Everard Home, we believe, had performed the operation some thirty times before any serious accident occurred, and an eminent surgeon, who, however, has wisely abandoned it, declared to us that he often had employed the ligature, and never, in his own practice, witnessed a bad result.

The testimony, or solitary experience, of any one man, in forming an estimate of the safety or danger of these operations on the veins, is positively dust in the balance. A surgeon may go on tying, or dividing, or cauterising, with impunity, till, at length, the day of reckoning arrives, and the death of his patient warns him, when too late, of his mischievous error. The general sense of the profession seems to be, to *operate* as little on the veins as possible; for, even when we do no harm, we do but little good, and the risk is out of all proportion to the benefit. If one venous trunk is choked up or destroyed, others rise, like the heads of the hydra, in its place, and, having placed the life of his patient in jeopardy, the surgeon too often discovers that, at last, he must trust to what he should have trusted to at first, compression and palliative measures.

---

